# A protocol for time-resolved transcriptomics through metabolic labeling and combinatorial indexing

**DOI:** 10.1016/j.xpro.2024.103356

**Published:** 2024-10-01

**Authors:** Rory J. Maizels, Daniel M. Snell, James Briscoe

**Affiliations:** 1 The Francis Crick Institute, 1 Midland Road, NW1 1AT London, UK; 2 University College London, London, UK

**Keywords:** Cell culture, Single Cell, Developmental biology, RNAseq, Molecular Biology, Gene Expression, Molecular/Chemical Probes

## Abstract

The snapshot nature of single-cell transcriptomics presents a challenge for studying the dynamics of gene expression. Metabolic labeling, where nascent RNA is labeled with 4-thiouridine (4sU), captures temporal information at the single-cell level, providing greater insight into expression dynamics. Here, we present an optimized, automation-friendly protocol for the metabolic labeling of RNA alongside single-cell RNA sequencing through combinatorial indexing. We describe steps for 4sU labeling, cell fixation and chemical treatment, and automated two-level combinatorial indexing.

For complete details on the use and execution of this protocol, please refer to Maizels et al.^1^

## Highlights

• sciFATE2 is a method for time-resolved single-cell transcriptomics
• 4sU labels nascent RNA in cells, capturing temporal information
• Iodoacetamide treatment converts 4sU to detectable cytosine analogs
• Automated combinatorial indexing captures 10,000 cells per experiment

## Abstract (editor highlights)

Publisher’s note: Undertaking any experimental protocol requires adherence to local institutional guidelines for laboratory safety and ethics.

## Graphical abstract

**Figure:**
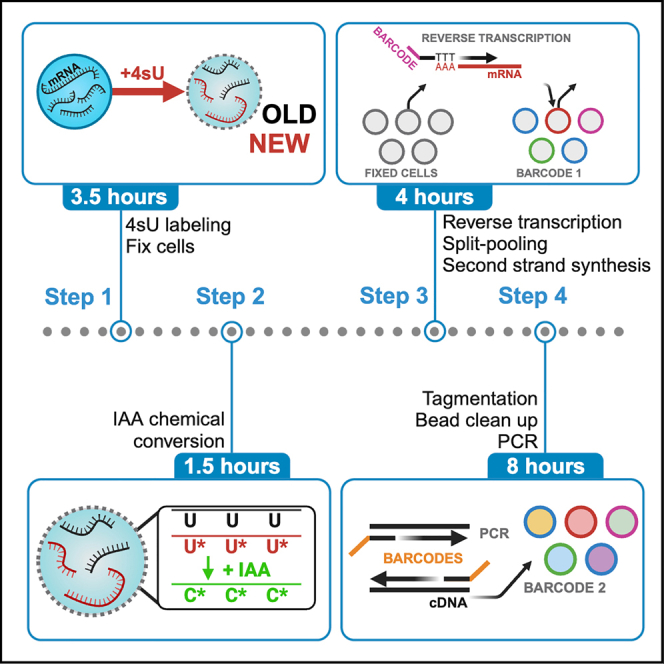

## Before you begin

**Figure 1:**
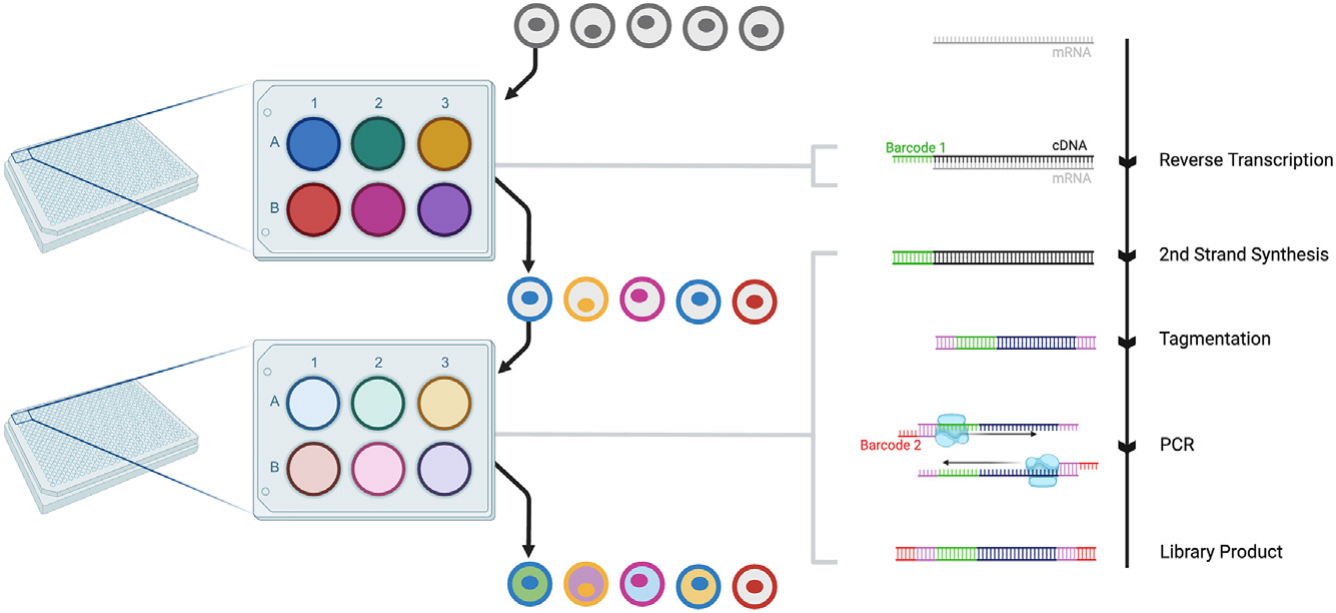
Overview of combinatorial indexing Cells are distributed into multi-well plates where they receive well-specific barcodes. Each individual barcode is given to multiple cells, but through the sequential incorporation of random barcodes, each individual cell should receive a unique combination of barcodes, providing single-cell resolution. For example, with two rounds of 384 barcodes, there are 147,456 possible barcode combinations, allowing over ten thousand cells to be sequenced per experiment with a ∼6% doublet rate. Reverse transcription is performed in the first plate with barcoded primers, then library preparation is completed in a second plate, with barcoded primers used for PCR amplification.

sciFATE2 is a protocol that combines metabolic labeling (incorporating 4-thiouridine, 4sU, into nascent RNA) with single-cell RNA sequencing through combinatorial indexing (Figure 1). This protocol describes the steps for carrying out sciFATE2 on an *in vitro* population of cells already grown to the desired stage; in the context of Maizels et al.,^1^ this was mouse embryonic stem cells put through an *in vitro* neural differentiation protocol.

### Preparing transposase

**Timing: 2 h**

***Note:*** Be sure not to confuse Tn5 dilution buffer (used to dilute the protein) and Tn5 buffer (used as actual reaction buffer)

***Note:*** Dimethylformamide (DMF) can dissolve some plastics; buffer should be prepared in a glass bottle on ice, and DMF should be measured out with a glass measuring cylinder.

1. Make Tn5 buffer.
  a. To the glass bottle on ice, add the following:

**Table:** 

Substance	Final concentration	Amount
Nuclease-free water	–	38.75 mL
1 M Tris-HCl pH 7.5	20 mM	1 mL
2 M MgCl_2_	10 mM	250 μL
Dimethylformamide	20% v/v	10 mL
**Total**	–	**50 mL**
  b. Mix well, make 1.25 mL aliquots and store at −20°C.
2. Make Tn5 dilution buffer (Note: different from Tn5 buffer).
  a. Add in a 2 mL Eppendorf tube:

**Table:** 

Substance	Final concentration	Amount
Nuclease-free water	–	800 μL
1 M Tris-HCl pH 7.5	50 mM	100 μL
5 M NaCl	100 mM	40 μL
10 mM EDTA (50-fold dilution of 0.5M stock in water)	0.1 mM	20 μL
100 mM DTT	1 mM	20 μL
10% IGEPAL CA-630	0.1% v/v	20 μL
Glycerol	50% v/v	1 mL
**Total**	–	**2 mL**
  b. Mix well and store at −20°C.
3. Resuspend the following DNA oligos at 100 μM in annealing buffer (50 mM NaCl, 40 mM Tris-HCl pH 8.0).
  a. Tn5-N7: 5′-GTCTCGTGGGCTCGGAGATGTGTATAAGAGACAG-3′.
  b. Tn5-ME: 5′-[Phos]CTGTCTCTTATACACATCT-3′.
4. Make adaptor mix.
  a. Mix 25 μL Tn5-N7 DNA oligo with 25 μL Tn5-ME DNA oligo.
  b. Run DNA oligo mix through the following thermocycler schedule:

**Table:** 

Temperature	Duration
95°C	5 min
Cool to 65°C	0.1 °C/s
65°C	5 min
Cool to 4°C	0.1 °C/s
Hold at 4°C	–

***Note:*** Annealed oligos can be frozen at −20°C, or directly processed to next steps.

5. Make transposase mix.
  a. Dilute Tn5 protein to 4 μM in Tn5 dilution buffer (not Tn5 buffer).
  b. Mix 20 μL of 4 μM Tn5 with 20 μL adaptor mix (prepared in step 4).
  c. Incubate at 23°C, 300 rpm for 30 min in a thermoblock.
  d. Add 20 μL glycerol, mix well.
  e. Store at −20°C.

### Making 4-thiouridine solution

**Timing: 10 min**

This is the stock solution used for metabolic labeling.

***Note:*** This should be done on the day of 4sU treatment. We have found that premade stocks of 4sU *can* be frozen and thawed at a later date, but because 4sU is sensitive to oxidation, we recommend making it fresh every time.

6. Make a 500 mM stock of 4-thiouridine (4sU) – this is a 1000X stock.
  a. Measure out dried 4sU and add 7.86 μL DMSO per mg of 4sU.
  b. Shake mix, protected from light, for 10 min, or until all solid 4sU is dissolved.

## Key resources table

**Table:** 

REAGENT or RESOURCE	SOURCE	IDENTIFIER
**Chemicals, peptides, and recombinant proteins**		

LIF	Merck	ESG1107
CHIR99021	Axon Medchem	1386
bFGF	PeproTech	100-18B
Retinoic acid	Merck	R2625
DAPI	Thermo Scientific	D1306
SAG	Calbiochem	566660
4sU	Sigma	T4509
DEPC	Sigma	D5758
SuperScript IV reverse transcriptase	Thermo Scientific	18090010
10 mM dNTP solution	NEB	N0447L
NEBNext high-fidelity 2X PCR master mix	NEB	M0541L
NEBNext Ultra II non-directional RNA second strand synthesis module	NEB	E6111L
Tn5 transposase	Produced in-house	N/A
Ampure XP beads	Beckman Coulter	A63881
Dimethylformamide	Fisher Scientific	10346180
Nuclease-free water	Thermo Scientific	AM9906
2 M MgCl_2_	Sigma	68475-100ML-F
DTT	Thermo Scientific	R0861
NEB albumin	New England Biolabs	B9200S
Iodoacetamide	Sigma	I6125-5G
Sodium phosphate buffer	Thermo Scientific	J61561.AP
DNA binding buffer	Zymo	D4003-1-25
IGEPAL CA-630	Sigma	I8896-50ML
RNase inhibitor	Thermo Scientific	10777019
Tris-HCl 7.5 / 8.0	Thermo Scientific	15567027 / 15568025
Dimethylformamide	Fisher Scientific	10590571
2 M MgCl_2_	Sigma/Merck	68475-100ML-F
EDTA 0.5 M	Thermo Scientific	AM9260G
Glycerol	Thermo Scientific	15514011
EB buffer	QIAGEN	19086

**Deposited data**		

Raw and processed sequencing data	This paper	GEO: GSE236520.

**Experimental models: Cell lines**		

HM1 mouse embryonic stem cells	Thermo Scientific	N/A

**Oligonucleotides**		

RT primer: ACGACGCTCTTCCGATCTNN NNNNNN[10-bp well-specific barcode] TTTTTTTTTTTTTTTTTTTTTTTTTTTTTTVN	Integrated DNA Technologies	Full list of primer oligos in Table S1
Tn5-N7: GTCTCGTGGGCTCGGAGATG TGTATAAGAGAC	N/A	N/A
Tn5-ME: [Phos]CTGTCTCTTATACACATCT	Integrated DNA Technologies	N/A
P5 PCR primer: AATGATACGGCGACCAC CGAGATCTACAC[i5]ACACTCTTTCCCTA CACGACGCTCTTCCGATCT	Integrated DNA Technologies	Full list of primer oligos in Table S1
P7 PCR primer: CAAGCAGAAGACGGCAT ACGAGAT[i7]GTCTCGTGGGCTCGG	Integrated DNA Technologies	Full list of primer oligos in Table S1

**Software and algorithms**		

sciFATE2_processing dynast	This paper	https://github.com/rorymaizels/sciFATE2_processing (https://doi.org/10.5281/zenodo.10815622)
Dynast	Qiu et al.^2^	https://dynast-release.readthedocs.io/en/latest/

**Other**		

Mosquito liquid handler	SPT Labtech	https://www.sptlabtech.com/products/mosquito
Magnetic plate holder	N/A	N/A
Magnetic tube rack	N/A	N/A
DNA LoBind tubes (1.5 mL / 2 mL)	Eppendorf	0030108051 / 0030108078
LoBind 384-well plates	Eppendorf	0030129547
LoBind 96-well plate	Eppendorf	0030129512
Thermo plate seals	Thermo Scientific	AB0558
Aluminum plate seals	VWR	391-1281

## Step-by-step method details

### 4sU labeling and cell fixation

**Timing: 3.5 h**

In this step, the cell population are labeled with 4-thiouridine (4sU) for a fixed time window (here presumed to be 2 h). Subsequently, they are collected and fixed with methanol. DEPC is included in the fixation step to inhibit RNases that can be released during the fixation process.

***Note:*** This protocol assumes the use of 500 μM 4sU for two hours. This was chosen based on the expected half-lives of dynamically expressed transcription factors and the number of previous studies that had successfully employed a two-hour labeling window.^2^^,^^3^ Both the concentration and duration of labeling can be altered subject to experimental optimization. Longer durations will lead to higher levels of incorporation and may be more suitable for studying the dynamics of genes with longer half-lives but can be toxic to cells. The effect of different labeling durations or concentrations on cellular behavior and function can be tested through any generic assay of cellular phenotypes, such as RNA-seq, RT-qPCR, immunofluorescence of key markers,^1^ while the level of incorporation of 4sU into RNA can be assessed either through sequencing or high performance liquid chromatography.^4^^,^^5^ To the authors’ knowledge, there are no cell types or media components that are explicitly incompatible.

***Note:*** The protocol should be generic across different cell types, but the authors have not extensively tested different cell types. Cell systems that develop significant three-dimensional structure may not be suitable, as this structure may affect diffusion of 4sU and introduce bias into label detection rates.

***Note:*** After labeling, we washed cells with PBS and media that did not contain any 4sU. This means that cells may be exposed to up to 10 min of 4sU-free media after labeling, which may add unlabeled nascent RNA to the cells’ transcriptomes. The degree to which this affects the data is not known, however future users may wish to include 4sU in these wash steps to ensure nascent RNA continues to be labeled.

***Note:*** The specific details of this preparation will depend on the experimental set-up and cell system. We used mouse embryonic stem cells differentiated *in vitro* to neural and mesodermal populations,^1^ but similar metabolic labeling approaches have been applied to a wide range of different cell types and systems.^2^^,^^3^^,^^5^

1. Culture cells so that they are ready to collect and sequence immediately after the labeling period.
2. Add 4sU to cells.
  a. Make cell media, with addition of 1:1000 500 mM 4sU stock for a final concentration of 500 μM.
  b. Replace cell media with 4sU containing buffer.
  c. Incubate for 2 h in the dark in normal conditions (for example, at 37°C and 5% CO_2_).
3. Prepare for cell fixation (immediately before collection).
  a. Make 1 M dithiothreitol (DTT) stock: add 155 mg DTT powder per 1 mL nuclease free water (alternatively Pierce no-weigh DTT can be used for ease).
  b. Make PBS-DTT: PBS + 1% 1M DTT.
  c. Make methanol-DTT-DEPC: methanol + 0.125% DEPC, 1% 1 M DTT.
  d. Prepare ice box for incubating samples during fixation.
4. Collect and fix cells.
  a. Wash cells with a volume of PBS equal to the volume of media they were contained in.
  b. Detach cells (for example: in a 35 mm well, with 200 μL 0.25% trypsin or accutase for 3 min; specific details depend on cell type).
  c. Collect cells in excess of the appropriate media for the cell system (e.g., add 1 mL media per 35 mm dish; using whatever media the cells were in during labeling).
  d. Count cells with hemocytometer.
  e. Spin cells at 1000 *g* for 3 m at room temperature.
  f. Resuspend cells at 5–25 M/mL in PBS-DTT, in 400 μL aliquots in 2 mL LoBind Eppendorf tubes.
    ***Note:*** The exact number of cells fixed per condition can be chosen based on experimental constraints and objectives. Fewer than 5 million cells may not give enough cells to complete the experiment, as the iodoacetamide treatment can lead to high cell loss. More than 25 million cells may start to reduce the efficacy of fixation, but this is not known.
  g. Add 1600 μL methanol-DTT-DEPC to the 2 mL Eppendorf tube Add the entire volume of methanol-DTT-DEPC to the tube slowly, dropwise. If necessary, during or after pipetting in the methanol, stir the liquid gently with the pipette tip to mix the cell suspension.
  h. Incubate tube on ice, gently rocking (20 rpm) for 30 min.
    **Pause point:** store tube(s) at −80°C for up to one month.

### Fixed cell treatment and iodoacetamide treatment

**Timing: 1.5 h**

At the beginning of the day of library preparation, the cell sample must be prepared and treated with iodoacetamide. This treatment converts incorporated 4sU into an analogue of cytosine that can be detected in sequencing.

**CRITICAL:** The number of aliquots of fixed cells used as input to the experiment can be varied based on experimental conditions and constraints. In general, a ≥1 mL suspension of ≥1 million cells (at 1 million/mL) is required. As much as 80% of cells can be lost during the iodoacetamide treatment steps. Thus, it may be advisable to start with 10 million cells. If multiplexing multiple conditions, it is still advisable to start with many millions of cells per condition. This is because the lower the starting number of cells, the higher the cell loss rate can be, and if too few cells are used (for example, 1 million or fewer), the entire sample can be lost. The rate of cell loss is also sensitive to the specific cell type: larger cell types, such as HEK293T cells, may have a lower cell loss rate than smaller cell types.

***Note:*** The protocol below is fixed regardless of number of cells or duration of 4sU, but we have not tested or optimized this extensively. If using a dramatically different number of cells or duration of labeling, it may be advisable to test different iodoacetamide treatment conditions.

5. Prepare reagents.
  a. Chill PBS on ice.
  b. 1 M DTT stock: add 155 mg DTT powder per 1 mL nuclease free water.
  c. PBSD: PBS + 0.1% DEPC + 3% NEB albumin + 1% 1 M DTT.
  d. PBSB: PBS + 3% NEB albumin.
  e. 100 mM iodoacetamide (IAA): 54 μL 100% ethanol per mg IAA. Dissolve on shaker, protected from light.
6. Move cell samples from −80°C to ice for 2–3 min (do this at least 2 h 45 min before flow sort).
7. Spin cells (1000 *g*, 3 min, 4°C), resuspend in 1 mL PBSD, spin again as before.

***Optional:*** Move cell sample to a 1.5 mL Eppendorf tube. This can help form a clearer pellet that is more robust to removal of supernatant.

8. Resuspend cells in 100 μL PBSB.
9. Add to cell suspension, while keeping on ice.
  a. 220 μL nuclease-free water.
  b. 40 μL 100 mM IAA.
  c. 40 μL sodium phosphate buffer.
10. Incubate at 50°C in a thermoblock for 15 min, gently flicking the tube every 5 min.

***Note:*** Whether longer durations would be beneficial when using longer 4sU labeling durations is not clear, however this incubation can be damaging to cells, so it may not be advisable to increase duration.

11. While waiting, prepare quenching mix in a 2 mL Eppendorf LoBind tube.
  a. 1.5 mL PBSB.
  b. 8.5 μL 1 M DTT.
12. After incubation, add cell sample to quenching mix tube.

***Note:*** Adding sample to quenching mix is done because sample is in a 1.5 mL tube, not a 2 mL tube. If cell sample remains in a 2 mL Eppendorf, quenching mix can be added to cell sample tubes, which may be quicker in the case of having multiple samples.

13. Spin cells down (1000 *g*, 3 min, 4°C). Resuspend cells at a concentration of 1 M/mL in PBS.
14. Proceed immediately to reverse transcription.

### Combinatorial indexing day one: Reverse transcription, split-pooling, second strand synthesis

**Timing: 4 h**

Here, cells are distributed into the first plate of combinatorial indexing, where reverse transcription with barcoded primers is performed. Then, cells are pooled, and flow sorted, 50 cells per well, into a second plate. In this plate, second strand synthesis is performed, then plates can be stored before continuing. The barcoded oligo-dT primer sequence contains the target sequence for PCR, an 8 bp UMI, a 10 bp barcode, and the oligo-dT sequence: ACGACGCTCTTCCGATCTNNNNNNNN[10-bp well-specific barcode]TTTTTTTTTTTTTTTTTTTTTTTTTTTTTTVN.

***Note:*** The protocol described here is designed to be performed with liquid handling robots; in our implementation, we used SPT Labtech Mosquito. The protocol should be possible to perform manually. However, this would be labor intensive and would be easier with two or more people.

15. Perform reverse transcription (start at least 1 h 15 min before flow sort).
  a. Make first-strand reaction mix (FSRM) per sample.
    i. 560 μL 5X SuperScript IV buffer.
    ii. 140 μL DTT (optional, can be replaced with water).
    iii. 140 μL RNase inhibitor (optional if DEPC was used, can be replaced with water).
    iv. 140 μL SuperScript IV reverse transcriptase.
    v. 140 μL 10mM dNTP mix.
  b. Add 2 μL of cells to each well of 384-well plate.
    ***Note:*** Using Mosquito automation, add >120 μL of cells to each well of a column of a 96-well plate, and distribute into plate.
  c. Add 1 μL of 25 μM barcoded oligo-dT primer to each well.
  d. Incubate plate at 55°C for 5 min, then place plate on ice for 2 min.
  e. Add 2 μL of FSRM to each well of the 384-well plate.
    ***Note:*** If using Mosquito automation, add 140 μL to each well of a column of a 96-well plate, and distribute into plate.
  f. Add plate to thermocycler and follow the following schedule:

**Table:** 

Temperature	Duration
4°C	2 min
10°C	2 min
20°C	2 min
30°C	2 min
40°C	2 min
50°C	2 min
55°C	10 min
Hold at 4°C	–
16. Perform split-pool step.
  a. Manually pool all cells from the 384-well plate.
    ***Note:*** To do this, use a multichannel pipette to pool every well of the 384-well plate into two columns (16 wells) of a 96 well plate. From here, pool all cells into a 2 mL Eppendorf tube. With the multichannel pipette, mix cell suspension a few times to dislodge cells from the plate before collecting up the cell suspension.
  b. Add 1.8 μL DAPI (1 mg/mL).
  c. Move cells to a FACS tube, passing through a cell strainer (40 μM for our cells) to remove aggregates. Add 200 μL NEBNext second strand synthesis buffer to 1,800 μL water and distribute this mix 4 μL per well into four 96-well LoBind plates.
  d. Flow sort cells into second-strand synthesis 96-well plates, 50 cells per well.
    ***Note:*** Gate using DAPI and scatter to remove debris and doublets. Typically, with our gates, 5% of events passed all gates to be sorted.
    ***Note:*** For our experiments, we used either the BD FACSAria Fusion or the MoFlo XDP. Ensure flow sorter is well calibrated to distribute cells to the center and bottom of plates. Keep plate and cells on ice before and after sorting.
17. Perform second-strand synthesis.
  a. Make SSS mix.
    i. 428 μL nuclease-free water.
    ii. 66 μL NEBNext second strand synthesis reaction buffer.
    iii. 165 μL NEBNext second strand synthesis enzyme.
  b. Add 1 μL to each well. If using Mosquito automation, add 82.5 μL per well of a column of a 96-well plate, and distribute into 384-well plate.
  c. Incubate cells for 3 h at 16°C.
  d. Plates can be kept overnight at 4°C, or at −20°C for longer durations.

***Note:*** If storing at −20°C, seal plates with aluminum not plastic seals.

### Combinatorial indexing day two: Tagmentation, PCR

**Timing: 6 h**

Here, plates are put through tagmentation, followed by purification by SPRI (Solid Phase Reversible Immobilization), then PCR with barcoded primers is performed. To ensure high-quality tagmentation reactions and bead clean ups, this is performed two plates at a time, in 96-well plate format, distributing the elution from bead clean ups back into a 384-well plate for PCR. However, with sufficient confidence or the necessary automation, this could be performed in a single 384-well run. The PCR primers used are of the sequences. P5 Primer: AATGATACGGCGACCACCGAGATCTACAC[i5]ACACTCTTTCCCTACACGACGCTCTTCCGATCT where i5 is the 10bp i5 barcode for illumina demultiplexing. P7 primer: CAAGCAGAAGACGGCATACGAGAT[i7]GTCTCGTGGGCTCGG where i5 is the 10bp i7 barcode for illumina demultiplexing. 384 i5/i7 indexes are used to provide cell barcoding.

**CRITICAL:** Tagmentation can be sensitive to varying input material, meaning that different cell types or number of cells may require different concentration of tagmentation enzyme Tn5 to ensure a high-quality library is produced.

***Note:*** Tagmentation can be optimized by running the sciFATE2 protocol as normal up until this step, then adding different Tn5 concentrations to different sections of the plate, pooling these sections separately after PCR, and inspecting the BioAnalyzer/Tapestation curves for each concentration. Generally, 32 wells or more per condition is sufficient such that, after concentration through SPRI, a clear library curve can be observed on the BioAnalyzer/Tapestation.

***Note:*** For tagmentation, we used Tn5 produced and purified in-house, as described previously.^1^^,^^6^ Commercial Tn5 can be used (the most cost-effective option that the authors are aware of is Active Motif 81286. Alternatively, Diagenode C01070010-500 can be used). However, it is important to note that because of the configuration of adaptor sequences used in this protocol, standard pre-loaded Tn5 from Illumina is not appropriate.

***Note:*** The number of PCR cycles used may require optimization for different cell types. For example, cell lines such as HEK293T may have a larger total amount of RNA per cell than other cell types, leading to more cDNA produced, requiring fewer PCR cycles. However, to ensure that correct library fragments (containing forward and reverse primers) sufficiently outnumber incorrect fragments (for example, containing two pairs of forward primers), it is suggested that eight to ten PCR cycles are performed.

18. Perform steps a-k twice, with two 96-well plates at a time (∼1.5 h each).
  a. Make Tn5 mix.
    i. 1170 μL Tn5 buffer (*not* Tn5 dilution buffer).
    ii. 30 μL Tn5.
  b. Add 5 μL Tn5 mix to each well. If using Mosquito automation, add 150 μL per well to a column of a 96-well plate and distribute.
  c. Incubate plate at 55°C for 5 min. Ensure DNA binding buffer is ready to add before the end of incubation to ensure a prompt end to tagmentation reaction.
  d. Add 10 μL DNA binding buffer to each well. Vortex plate at ∼800 rpm for 30 s on a plate vortexer and incubate at room temperature for 5 min.
  e. Add 30 μL Ampure XP beads per well. Vortex plates at ∼800 rpm for 30 s on a plate vortexer. Incubate at room temperature for 10 min.
  f. Add plates to magnetic plate holder, aspirate 40 μL of supernatant.
  g. Add 100 μL 80% ethanol, aspirate all liquid.
  h. Repeat ethanol wash in step g.
  i. Air dry plates for ∼2 min.
  j. Add 10 μL EB buffer to each well. Vortex plates thoroughly to ensure proper mixing.
  k. Incubate for 10 min at room temperature.
  l. Return plates to magnetic plate holder, move 9 μL from each well to half of a 384-well plate.
  m. Repeat with third and fourth plates, moving to other half of 384-well plate.
19. Run PCR.
  a. Add 1 μL PCR primer mix per well.
  b. Add 10 μL PCR mix to each well.
  c. Run PCR according to the following schedule:

**Table:** 

Step	Cycle step	Duration
72°C	–	5 min
98°C	–	30 s
10 cycles:	–	–
–	98°C	10 s
–	66°C	30 s
–	72°C	1 min
72°C	–	5 min
Hold at 4°C	–	–

### Clean up & final steps

**Timing: 2 h**

With library preparation complete, the final library must be cleaned and concentrated through SPRI bead clean up.

20. Pool entire library.
  a. Pool with multi-channel pipette into 4 rows of a 96-well plate.
  b. Pool further into 8 × 1.5 mL LoBind Eppendorf tubes.
21. Perform first clean up.
  a. Add 765 μL Ampure XP beads to each sample (0.9X volume). Mix thoroughly, incubate at room temperature for 5 min.
  b. Add tubes to magnetic tube rack, remove liquid leaving a small amount (∼50 μL) behind.
  c. Add 1.5 mL 80% ethanol, aspirate entirely off.
  d. Repeat ethanol wash in step c.
  e. With aspirator, remove any excess ethanol from the inside of the tube, ensuring not to touch the bead pellet.
  f. Remove tubes from magnetic rack. Air dry the pellet until no longer shiny; if pellet starts to crack, proceed to next step immediately.
  g. Add 102 μL of EB buffer to each tube. Mix thoroughly and incubate at room temperature for 10 min.
  h. Return tubes to magnetic rack, move 100 μL from each tube into 2 tubes, 400 μL each.
22. Perform second, double-size selection clean up.
  a. Add 200 μL Ampure XP beads (0.5X volume) to each tube. Mix thoroughly, incubate for 10 min at room temperature.
  b. Add to magnet, and move 600 μL from each tube to new tubes, leaving beads behind.
  c. Add 160 μL AMPure XP beads to each tube, bringing SPRI buffer volume up to 360 μL (0.9X initial volume), mix thoroughly and incubate for 5 min.
  d. Add to sample to magnetic tube rack, aspirate liquid, leaving a small volume (∼10 μL).
  e. Add 1 mL 80% ethanol, aspirate entirely off.
  f. Repeat ethanol wash in step c.
  g. Remove tubes from magnetic rack. Air dry the pellet until no longer shiny; if pellet starts to crack, proceed to next step immediately.
  h. Add 32 μL EB buffer to both, mix thoroughly, incubate for 10 min at room temperature.
  i. Collect 30 μL from both tubes into a single final tube of 60 μL.
23. Run Qubit and BioAnalyzer/Tapestation QC analysis to quantify library size and quantification: no dilution should be necessary for this.
24. Submit to sequencing. For sciFATE2 libraries, we sequenced 700M reads per library with the following configuration: (read 1: index 1: index 2: read 2): 18-10-10-130.

## Expected outcomes

**Figure 2:**
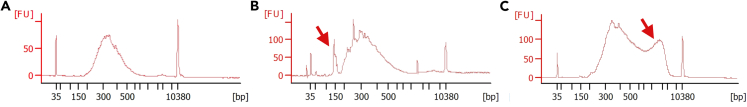
Examples of BioAnalyzer traces of final library products (A) A suitable BioAnalyzer trace. (B) A BioAnalyzer trace showing primer-dimer contamination (red arrow). (C) A BioAnalyzer trace showing high molecular weight contamination, from overamplification or genomic DNA contamination.

The outcome of this protocol should be 60 μL of library sample, with a concentration of at least 1 ng/μL and an average library size of around 300–500 bp (Figure 2A). This should ideally be assessed using Qubit and BioAnalyzer/Tapestation. There should be minimal to no primer dimers in the mix; if there is, a sharp peak around 150 bp will be observed (Figure 2B). There should be minimal to no high molecular weight contaminant in the mix (caused by genomic contamination and/or overamplification); if there is, a broad flat peak above 1000 bp will be observed (Figure 2C). If the final library contains either low or high molecular weight contamination, the final double size selection with AMPure beads can be repeated (with an initial 0.5X volume added, then an addition 0.4X volume added for second step). See Troubleshooting 1, 2, 3, 4, and 5 for more details.

## Quantification and statistical analysis

After sequencing, samples from each PCR well should be demultiplexed based on Illumina indices into separate fastq files. For these fastq files, read 1 will contain the UMI and cell barcode information, read 2 will contain the sequence information. Downstream processing of fastq files can be performed using a custom pipeline that implements Dynast.^4^ This pipeline can be found here: https://github.com/rorymaizels/sciFATE2_processing.

The output of this will be an Anndata object for each PCR sample, which can then be collected into a single Anndata object for downstream analysis. Running sciFATE2 on neural progenitors, we observed over ten thousand cells per experiment (using a ‘cell’ threshold of >1000 UMIs), with a median of between 10,000 and 20,000 UMIs per cell from around 70,000–80,000 sequencing reads per cell.

**Figure 3:**
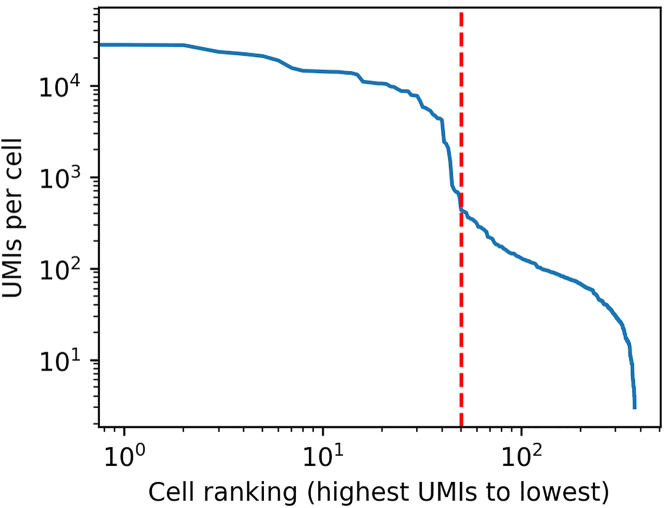
UMIs per barcode/cell, ordering barcodes from highest to lowest UMIs, shown on a log-log scale A sharp elbow shows the transition from real cellular content to ambient RNA, occurring just before the theoretical maximum number of cells, fifty (red dotted line).

Even with 50 cells per well, it is normal to see many more barcodes in each PCR sample, due to ambient RNA. However, sorting barcodes in descending order of UMIs and plotting UMIs per cell/barcode against cell ranking on a log-log scale should reveal a clear elbow/threshold that should drop before the theoretical limit of 50 cells per well (Figure 3).

Raw and processed data from existing sciFATE2 experiments can be accessed from Gene Expression Omnibus with accession GEO: GSE236520.

## Limitations

While metabolic labeling has be applied *in vivo*, the rate of distribution and uptake of 4sU in three-dimensional tissues may vary considerably between cell types, biasing the dynamics that are recorded. In this protocol, we used 2 h of 4sU labeling at a concentration of 500 μM in neural progenitor populations; we confirmed that this labeling regime did not induce any observable cellular effects through RT-qPCR, immunofluorescence microscopy, RNA-seq and cell viability assays, but moving to a new cell system, longer labeling duration or higher concentration of 4sU may require more tests to confirm that toxicity is not observed.

Combinatorial indexing assumes that input cells exist in a single-cell suspension: if fixation causes clumping of cells, this can distort data and lead to a high rate of doublets; thus, the effect of fixation on different cell types must be carefully examined.

This protocol is implemented using liquid handling robots in numerous steps; the protocol is possible to do manually, but very labor intensive for a single researcher to perform alone. If performing the protocol manually, it would be advisable to do so with two or more people at once.

## Troubleshooting

### Problem 1

The peak of the library is too large (e.g., above 600 bp) in quantification (step 24).

### Potential solution

This can be caused by under-tagmentation. Increase the concentration of Tn5 used in reactions.

### Problem 2

There is a second, larger peak in my BioAnalyzer trace in quantification (step 24).

### Potential solution

This could have various causes. Try: increasing Tn5 concentration, running fewer PCR cycles, running a more stringent double-size SPRI bead clean-up to remove genomic DNA contamination. If you observe this *after* bead clean up, it can be helpful to visual libraries through BioAnalyzer/Tapestation after zero, one and two clean ups.

### Problem 3

There are primer-dimer low-molecular weight peaks even after clean-up in quantification (step 24).

### Potential solution

While this could be caused by inefficient bead clean up, it can also be caused by failed library preparation, as we have observed that the lack of a library of suitable size (>200 bp) reduces the ability of SPRI bead cleaning to remove smaller fragments from the sample. Failed library amplification also reduces the depletion of primer pools, leading to more likely primer-dimer contamination.

Additional or more stringent bead cleaning could solve this issue, but optimizing the protocol to ensure library preparation is successful (for example, ensuring that somewhere in the region of 50 ng of library DNA is being produced) may be required.

### Problem 4

There is little or no library at quantification (step 24).

### Potential solution

This can have many causes and may require careful optimization. It is not possible to be exhaustive in suggesting solutions, but there are some key ways to assess the protocol.

• Use BioAnalyzer RNA integrity evaluation to assess the quality of RNA after cell fixation.
• Try the protocol with a standard cell line like HEK293T or A549 cells, as these lines have a large amount of transcriptomic content.
• Perform targeted qPCR of key genes known to be expressed in the cell systems to assess independently if reverse transcription is working.
• Test Tn5 on genomic DNA (directly inserting P5 and P7 handles, rather than just P7 handle, as if doing ATAC-seq) to assess if the tagmentation process is working.
• Check the pipetting works properly for each step, particularly low-volume steps like second-strand synthesis.
• Before PCR, run a qPCR of sample using the same primers, and see how many cycles are needed for efficient amplification (the ideal amount of amplification would be 10%–25% maximum).

### Problem 5

The library quantification through Qubit and BioAnalyzer, but when I sequence the library, I get under-clustering or poor data QC.

### Potential solution

BioAnalyzer and Qubit only assess the amount and size of DNA fragments, not whether they are correctly amplifiable. For this, one can use library quantification through qPCR (KAPA). Under-clustering can be caused by insufficient PCR amplification: because tagmentation creates many linearly amplifiable products throughout the cDNA, and only one exponentially amplifiable product at the 3′ end of the cDNA, sufficient PCR amplification is required to dilute out linearly amplified products. It is not recommended to do fewer than 8, and ideally 10 PCR cycles. If there are contaminants in the mix that inhibit PCR or reduce PCR efficiency, this can also lead to underclustering through the same mechanism.

### Problem 6

I want to test the protocol before committing to purchasing primer plates.

### Potential solution

It is possible to test the protocol without single-cell resolution to approximate data quality. For this, an unbarcoded reverse transcription primer can be used, if it still has a UMI sequence. In PCR, a small number of barcoded primers can be used to provide greater resolution on data quality without full single cell. For example, with 16 PCR primers, each barcode can represent 24 wells. If 50 cells are added per well, one can expect around 30 high quality transcriptomes per well, which translates to 720 cells per barcode. Dividing the number of UMIs observed per barcode by this estimated cell number can provide a rough approximation of data quality.

## Resource availability

### Lead contact

Further information and requests for resources and reagents should be directed to and will be fulfilled by the lead contact, James Briscoe, james.briscoe@crick.ac.uk.

### Technical contact

Technical questions on executing this protocol should be directed to and will be answered by the technical contact, Rory Maizels, rory.maizels@crick.ac.uk.

### Materials availability

Barcoded primers used in combinatorial indexing available as supplementary file. All other materials are commercially available.

### Data and code availability

The accession number for the raw sequencing data and processed sequencing data generated using sciFATE2 as reported in this paper is GEO: GSE236520. All original code for raw data processing and label detection with dynast is available at https://github.com/rorymaizels/sciFATE2_processing. All original code for computational analysis using sciFATE2 data is available at https://github.com/rorymaizels/Maizels2023aa. A deep learning framework, available as the software package, velvetvae, for modeling dynamics from sciFATE2 data, can be installed from https://github.com/rorymaizels/velvetvae. Any additional information required to reanalyze the data reported in this paper is available from the lead contact upon request.

## Acknowledgments

We are grateful to the Advanced Sequencing STP and Flow Cytometry STP at the Francis Crick Institute for their assistance in carrying out experiments and to the Structural Biology STP for the production of Tn5. This work was supported by the Francis Crick Institute, which receives its core funding from Cancer Research UK (CC001051), the UK Medical Research Council (CC001051), and the Wellcome Trust (CC001051); by the European Research Council, under European Union (EU) Horizon 2020 research and innovation program grant 742138; and by the Wellcome Trust (220379/D/20/Z). For the purpose of open access, the author has applied a CC BY public copyright license to any author-accepted manuscript version arising from this submission. Figures were produced with biorender.com.

## Author contributions

R.J.M. and J.B. conceived the project, interpreted data, and wrote the manuscript. R.J.M. designed and performed the experiments and developed computational methods for downstream processing and data analysis. R.J.M. and D.M.S. developed and optimized automation of the experimental protocol.

## Declaration of interests

R.J.M. is a consultant for Omnipotent Biotechnologies.

## Supplemental information

Table S1. List of barcoded primers used for sciFATE2, used for steps 16c and 20a
